# Gastric remnant fundoplication for refractory GERD post-roux-en-Y gastric bypass: A case report

**DOI:** 10.1016/j.ijscr.2025.111523

**Published:** 2025-06-16

**Authors:** Rakan Mal, Ahmad Jan, Mohsin Yahya Murshid, Badr Beyari, Munirah Fetaini

**Affiliations:** a Department of General Surgery, King Abdulaziz Hospital, Jeddah, Saudi Arabia; b Department of General Surgery, International Medical Center, Jeddah, Saudi Arabia; c Department of General Surgery, Hera General Hospital, Makkah, Saudi Arabia

**Keywords:** Roux-en-Y gastric bypass (RYGB), Refractory GERD, Fundoplication, Gastric remnant, Revisional surgery, Case report

## Abstract

**Introduction and importance:**

Gastroesophageal reflux disease (GERD) commonly affects individuals with obesity, often worsened by increased intra-abdominal pressure and hiatal hernias. Roux-en-Y gastric bypass (RYGB) is preferred for obese patients with GERD due to its effectiveness in weight loss and symptom relief, but some patients experience persistent or recurrent GERD post-surgery. In cases resistant to medical treatment, additional surgical intervention may be required. This case report described using the gastric remnant for fundoplication in patients with refractory GERD after RYGB.

**Case presentation:**

A 48-year-old female with obesity and GERD underwent RYGB with concurrent hiatal hernia repair. Despite initial improvement, she developed treatment-resistant GERD. Endoscopy revealed esophagitis and bile reflux. Due to the severity of her symptoms, a revisional surgery was performed using the gastric remnant for fundoplication, leading to complete symptom resolution postoperatively.

**Clinical discussion:**

Managing GERD post-RYGB is challenging when medical therapy fails. Traditional fundoplication is unfeasible due to the absence of a gastric fundus. Alternative approaches, such as Hill's repair, ligamentum teres cardiopexy, and magnetic sphincter augmentation, have uncertain long-term efficacy. Gastric remnant fundoplication offers a promising solution by restoring the anti-reflux mechanism while preserving bypass anatomy.

**Conclusion:**

Refractory GERD after RYGB is a significant challenge. Gastric remnant fundoplication effectively controls reflux while maintaining bypass integrity. This report presents the first documented use of this technique in Saudi Arabia. Further research is needed to evaluate long-term outcomes and establish standardized guidelines.

## Highlights

• Refractory GERD may persist or recur even after Roux-en-Y gastric bypass (RYGB).
• Traditional fundoplication is not feasible post-RYGB due to altered anatomy.
• Gastric remnant fundoplication is a novel, anatomy-preserving solution.
• This is the first reported case of this technique in Saudi Arabia.
• The procedure resulted in full symptom resolution within 3 months.

## 1 Introduction

Gastroesophageal reflux disease (GERD) is a prevalent condition among individuals with obesity, primarily due to increased intra-abdominal pressure and the accumulation of intra abdominal fat. These factors elevate intragastric pressure, alter the gastro-esophageal gradient, and displace the lower esophageal sphincter, leading to heightened esophageal exposure to gastric contents [1]. A review indicates that obesity is linked to a 1.5- to 2-fold higher risk of experiencing GERD symptoms and erosive esophagitis, as well as a 2- to 2.5-fold increased risk of developing esophageal adenocarcinoma compared to individuals with a normal BMI [2]. Additionally, hiatal hernia, which is highly prevalent among obese individuals, has been shown to significantly increase the risk of GERD symptoms [3].

Bariatric surgery is the most effective long-term intervention for obesity, with the choice of procedure often influenced by the presence of persistent GERD. Roux-en-Y gastric bypass (RYGB) is commonly recommended for patients with concurrent obesity and GERD. However, studies have reported varying prevalence rates of persistent GERD post-RYGB, ranging from 1 % to 18 % over periods between 2 and 5 years [4]. Furthermore, it has been reported that about one-third of patients who had GERD before surgery still required anti- reflux medications 10 years postoperatively [5].

In this case report, we present a 48-year-old female patient with a history of morbid obesity, hiatal hernia, and GERD symptoms. Despite undergoing RYGB and hiatal hernia repair, her GERD symptoms recurred after a period of remission and were refractory to medical therapy. We are reporting a surgical approach utilizing the gastric remnant for fundoplication, which successfully resolved her persistent GERD. This report has been prepared in accordance with the updated consensus Surgical CAse REport (SCARE) guidelines [6].

## 2 Case presentation

A 48-year-old female patient with a BMI of 39 kg/m^2^ and history of obesity-related disorders, including non-alcoholic fatty liver disease (NAFLD), hyperlipidemia, and multiple joint arthritis. She also complained of intermittent gastroesophageal reflux disease (GERD) symptoms. Preoperative evaluations revealed a 2–3 cm hiatal hernia and mid-esophageal reflux through an upper endoscopy and a barium swallow study, respectively. In October 2023, she underwent laparoscopic Roux-en-Y gastric bypass (RYGB) with concurrent hiatal hernia repair. Fundoplication was not performed at that time, as the hernia was addressed with crural approximation, and RYGB itself is considered an effective anti-reflux procedure; thus, additional reinforcement is not standard practice during initial hiatal hernia repair in such cases.

One-year post-surgery, in December 2024, despite her BMI reducing to 29 kg/m^2^, she experienced a resurgence of regurgitation, heartburn, and epigastric pain. A subsequent upper endoscopy showed Los Angeles Grade A esophagitis and bile reflux in the gastric pouch, confirming a normal pouch size, negative *Helicobacter pylori* status, and an intact anastomosis. Despite a six-month course of proton pump inhibitors (PPIs) and ursodeoxycholic acid, she continued to experience persistent GERD. Esophageal manometry was not performed at this stage because the patient had no symptoms suggestive of esophageal motility disorders, such as dysphagia or food sticking. The clinical picture was dominated by severe reflux, supported by endoscopic evidence of bile-stained fluid in the gastric pouch and a recurrent hiatal hernia. Given the low suspicion of a motility disorder and standard practice at our center, manometry was not indicated. Consequently, a decision was made to pursue a second surgical intervention to address her persistent GERD symptoms.

## 3 Surgical approach

The procedure was performed by a consultant laparoscopic and bariatric surgeon with substantial experience in upper GI surgery. His practice includes over 3000 laparoscopic Roux-en-Y gastric bypasses, numerous hiatal hernia repairs, and various anti-reflux procedures including revisional Nissen and Toupet fundoplications. This expertise supported the decision to adopt a tailored approach using the gastric remnant.

The patient was placed under general anesthesia and positioned in a modified lithotomy stance. Access to the abdomen was gained via a 10 mm trocar introduced at Palmer's Point, supplemented by a supraumbilical 10 mm trocar and two 5 mm trocars along the anterior axillary lines. Extensive adhesions between the liver and the stomach were encountered and carefully lysed. To facilitate exposure of the hiatus, a liver retractor was used to elevate the left dome of the liver. A peritoneal incision was made over the hiatus to expose the left and right crus. A circumferential dissection around the esophagus was performed, creating a retroesophageal window while preserving the vagus nerve. The esophagus was then encircled using a gold finger, with an esophageal bougie inserted through an orogastro-duodenoscopy (OGD).

**Fig. 1:**
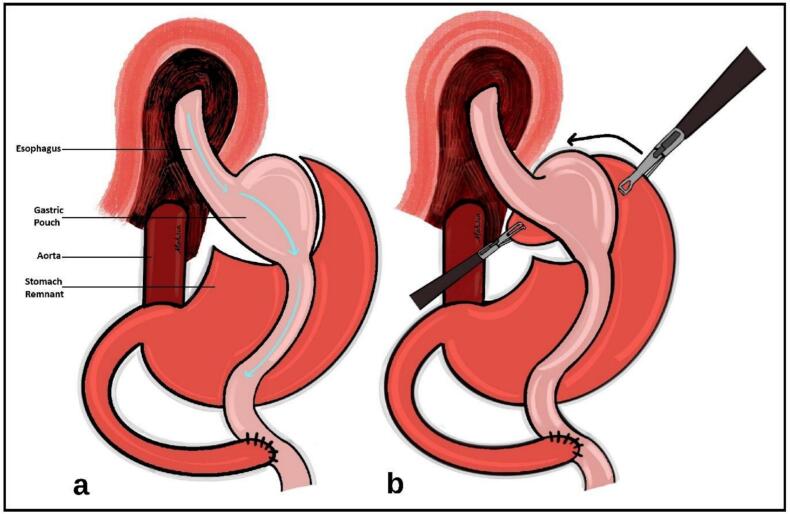
(a) Schematic illustration showing the altered gastrointestinal anatomy following Roux-en-Y gastric bypass, including the creation of a small gastric pouch and bypassed gastric remnant. (b) Schematic illustration showing the gastric remnant being mobilized and wrapped 360 degrees around the lower esophagus to form a fundoplication.

Given the small size of the gastric-roux-en-y pouch, a standard Nissen fundoplication was not feasible. Instead, the remnant of the stomach, which had been stapled and sealed off during the previous RYGB, was utilized to wrap the esophagus (Fig. 1).

**Fig. 2:**
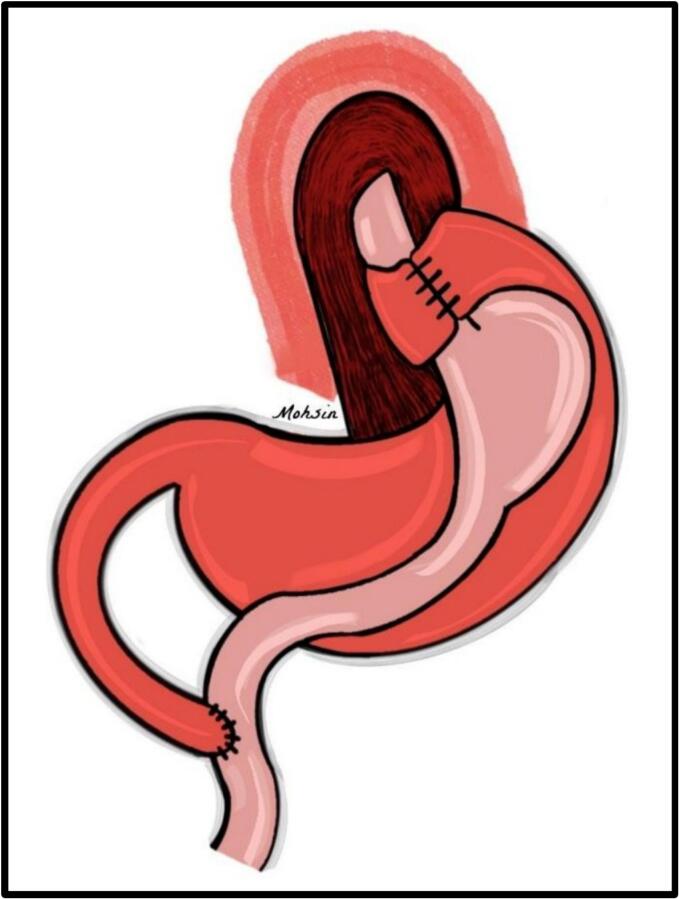
Schematic illustration depicting the completed 360-degree gastric remnant fundoplication. The wrap is secured with sutures, incorporating the anterior wall of the esophagus to ensure adequate tightness and restore the anti-reflux barrier.

This required initial adhesiolysis to release adhesions between the gastric-roux-en-y pouch and the gastric remnant. The gastric remnant was found adherent to the splenic hilum and its vessels and was approached from the lesser sac through a window on the greater curvature side using Ligasure. The proximal part of the stomach remnant was freed from the attached greater omentum for about 10 cm to prepare for the wrap. The vascularity of the stomach remnant was confirmed with indocyanine green (ICG), ensuring intact blood flow. ICG was administered at a dose of 3 mg, 30 min before the procedure and repeated one hour postoperatively. The fundoplication wrap was then passed behind the esophagus, covering approximately 5 cm. To ensure the wrap was sufficiently tight, a shoeshine maneuver was performed. The fundus was sutured to itself, including the anterior esophageal wall with four stitches (Fig. 2). Concurrently, a standard cholecystectomy was performed due to the presence of tiny multiple gallstones.

**Fig. 3:**
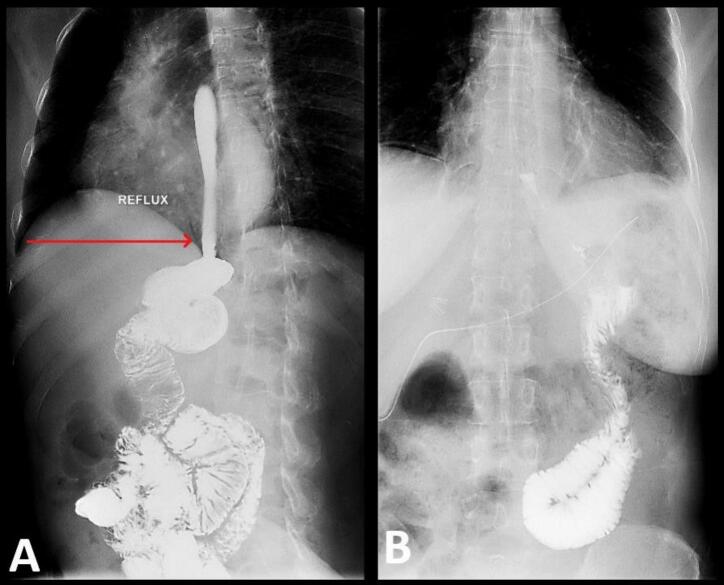
Fluoroscopic Study showing (A) Gastroesophageal Reflux. (B) Absence of reflux after fundoplication.

The total operative time was approximately 3 h, including adhesiolysis and fundoplication, with an estimated blood loss of less than 100 mL. Antibiotic prophylaxis included intravenous cefuroxime and Kilixan administered during induction. Pain management consisted of ibuprofen, oxycodone 10 mg, and Laproxin as needed. A contrast swallow fluoroscopic study was performed on the first post-operative day, no reflux was detected (Fig. 3). Patient was then discharged home and followed up in the clinic over the next 3 months. The Patient demonstrated full recovery and complete resolution of her GERD related symptoms, the patient was also satisfied with the outcome of this surgery.

To objectively assess symptom severity and treatment response, we used the Gastroesophageal Reflux Disease–Health-Related Quality of Life (GERD-HRQL) questionnaire preoperatively and 3 months postoperatively. The GERD-HRQL is a validated tool that quantifies symptom burden, with higher scores indicating more severe symptoms. The table below summarizes the patient's scores (Table 1).

**Table 1:** GERD-HRQL score comparison before and after gastric remnant fundoplication. The scores range from 0 (no symptoms) to 5 (incapacitating symptoms). The satisfaction score is reverse scored to align with severity grading.

GERD-HRQL symptom	Preoperative score	Postoperative score (3 months)
Heartburn when lying down	4	1
Heartburn when standing up	2	0
Heartburn after meals	4	1
Impact of heartburn on daily activities	4	0
Difficulty swallowing	1	0
Sensation of food sticking	1	1
Cough or hoarseness due to reflux	5	0
Taking medications for reflux	5	1
Satisfaction with current condition (reverse scored)	1	5
Sleep disturbance from reflux	5	1
Total Score (out of 50)	32	10

## 4 Discussion

Roux-en-Y gastric bypass (RYGB) is considered the optimal procedure of choice for treating morbid-obesity with associated GERD. This procedure contributes to GERD alleviation by facilitating rapid gastric emptying, reducing lower esophageal sphincter pressure, diverting bile, decreasing parietal cells through the exclusion of the gastric body, and aiding in weight reduction [7]. While one study reported an 80 % resolution of GERD symptoms following RYGB [8], others have highlighted varying outcomes. Some studies found GERD symptom prevalence rates ranging from 1 % to 18 % post-RYGB over 2 to 5 years [4], and another reported that about one-third of patients required ongoing anti-reflux medications 10 years after the surgery [5].

Challenges in managing GERD post-RYGB include surgical technicalities such as the persistence of acid-secreting parietal cells in the small gastric pouch and the restrictive nature of the gastrojejunal anastomosis, which may increase lower esophageal sphincter pressure and contribute to reflux [9]. Complications such as hiatal hernia and gastrogastric fistula can impair gastric emptying or allow acid passage. In this case, the patient had longstanding GERD prior to RYGB, complicating the determination of which surgical factors might have influenced her postoperative reflux.

Recurrent hiatal hernia has also been implicated in the onset of GERD post-RYGB, with one series suggesting that difficult emptying of the herniated stomach contributes to distal esophageal damage, particularly when the hernia exceeds 4 cm [10]. However, despite the repair of her initial hiatal hernia during RYGB, our patient continued to experience persistent GERD. Subsequent upper endoscopies revealed a recurrent hiatal hernia of less than 2 cm, lacking a sliding component, making it an unlikely sole contributor to her reflux, thus necessitating further surgical intervention.

Moreover, other typical post-RYGB risk factors for GERD such as a large pouch, active gastritis, and tight anastomosis were ruled out during the preoperative evaluation. However, the functionality of the lower esophageal sphincter was not assessed prior to the repeat surgery, leaving unanswered questions about its role in the patient's recurring symptoms. The pause in GERD symptoms between the two surgeries suggests that the causes of her GERD were not consistent across both incidents, supported by her non-obese status and the small hiatal hernia before the second procedure.

Several surgical options to manage GERD post RYGB are documented, including Hill's repair, ligamentum teres cardiopexy, magnetic sphincter augmentation with the LINX system, and fundoplication using the gastric remnant [9] (Table 2). However, each alternative presents significant limitations in the post-RYGB setting. For example, the LINX device has not been validated for use in patients with altered foregut anatomy. The U.S. FDA approval specifically excludes individuals with prior foregut surgeries like RYGB due to concerns about device efficacy and safety in such populations [11]. Furthermore, the Hill repair technique relies on anatomical structures such as the gastric cardia and angle of His, both of which are anatomically bypassed and excluded in RYGB patients, rendering the procedure technically infeasible. In this case, we employed fundoplication with the gastric remnant, a technique previously described by various surgeons and reported to maintain the esophagus in an intra-abdominal position, thus reducing the distensibility and increasing the length of the lower esophageal sphincter [9]. This approach provides a continuous anti-reflux mechanism, unlike traditional fundoplication, which relies on gastric filling to compress the sphincter [9]. A 360-degree fundoplication also diminishes transient LES relaxations by limiting gastric cardia distension, thus reducing reflux episodes [12].

**Table 2:** Comparison of possible surgical options for refractory GERD post RYGB.

Procedure	Approach	Advantages	Disadvantages	Clinical outcomes	Evidence level
Hill's Repair	Anchors esophagus to median arcuate ligament; reinforces phrenoesophageal membrane	Restores natural anatomy; may reduce reflux	Technically demanding; limited surgeon experience	Limited data on success/recurrence; further studies needed	Case series
Ligamentum Teres Cardiopexy	Uses ligamentum teres to buttress gastroesophageal junction	Autologous tissue; reduces foreign body risks	Limited experience; uncertain long-term efficacy	Outcomes not well-documented due to novelty	Case series
Magnetic Sphincter Augmentation (LINX)	Places magnetic beads around LES to augment pressure	Minimally invasive; preserves anatomy; physiological reflux control	Risk of device erosion/migration; may interfere with MRI	Promising short- term results; limited long-term data	Systematic review and meta-analysis
Fundoplication with Gastric Remnant	Uses gastric remnant to create fundoplication wrap around esophagus	Robust anti-reflux barrier; no foreign materials	Technically complex due to adhesions/altered anatomy	Initial success reported; long- term outcomes/recurrence rates unclear	Case series

Our patient represents the first documented case in Saudi Arabia to undergo this procedure, with complete resolution of symptoms, suggesting an effective rescue option for refractory GERD post-RYGB. Similar success has been reported in the literature. For instance, Vorwald et al. described a patient who remained symptom-free one year after undergoing laparoscopic Toupet fundoplication following RYGB, with normal pH monitoring and radiologic findings [13]. Likewise, Kawahara et al. reported favorable outcomes using modified Nissen fundoplication post-RYGB without symptom recurrence during follow-up [14]. These findings support the potential efficacy of remnant-based fundoplication procedures in carefully selected patients.

Nonetheless, the procedure is not without risks. A review of revisional anti-reflux surgeries after RYGB noted a complication rate of 16.7 %, with issues including anastomotic leaks and strictures [15]. In our case, no such complications were observed, likely due to meticulous dissection and preoperative planning. A literature search was conducted using PubMed and Google Scholar with relevant keywords (e.g., “gastric remnant fundoplication,” “Roux-en-Y gastric bypass,” “Saudi Arabia”), and no prior reports were identified, supporting the novelty of this case. The success aligns with earlier studies [14,[16], [17], [18]] and highlights the benefits of this innovative surgical strategy in the post-RYGB patient population.

## 5 Conclusion

Roux-en-Y gastric bypass (RYGB) is a prevalent bariatric surgery renowned for its efficacy in managing obesity and gastroesophageal reflux disease (GERD). However, as demonstrated in our case and corroborated by other studies, GERD can persist or recur post-RYGB in some patients. When medical therapy is insufficient, it becomes essential to explore effective surgical alternatives. Fundoplication using the gastric remnant has emerged as an outstanding rescue procedure for refractory GERD following RYGB, providing symptom relief and a robust anti-reflux mechanism compared to other modalities. A key limitation of this report is the short follow-up duration (3 months), which limits our ability to assess the long-term durability of symptom resolution and potential complications. Future studies with longer follow-up periods and larger patient cohorts are essential to validate the efficacy and safety of gastric remnant fundoplication in post-RYGB patients. Further prospective studies are imperative to assess the long-term outcomes and to formulate standardized surgical protocols.

## Abbreviations

GERD: Gastroesophageal Reflux Disease
PPIs: Proton Pump Inhibitors
RYGB: Roux-en-Y Gastric Bypass
OGD: Orogastro-duodenoscopy
BMI: Body Mass Index
ICG: Indocyanine Green
NAFLD: Non-Alcoholic Fatty Liver Disease
LES: Lower Esophageal Sphincter
MRI: Magnetic Resonance Imaging
SCARE: Surgical CAse REport

## Consent of publication

Written informed consent was obtained from the patient.

## Ethical approval

This study was approved by IRB committee of the research center of International Medical Center (IMC). Ethical approval number (IMC-IRB #: 2025–04-273).

## Funding

None.

## Author contribution

Rakan Mal, first author, major writer.

Ahmed Jan, second and corresponding author, he is the surgeon.

Mohsin Murshid, third author, did proofreading and graphics designer.

Badir Bayari, fourth author, proofreading.

Monira Futaini, fifth author, proofreading.

## Guarantor

Rakan Mal.

## Research registration number

N/A.

## Conflict of interest statement

The authors declared that there is no conflict of interest regarding the publication of this case report.
